# Future Problem-Solving Practiced During COVID-19: Implications for Health Management Students' E-Health Literacy Identity

**DOI:** 10.3389/fpsyg.2022.829243

**Published:** 2022-02-17

**Authors:** Dorit Alt, Lior Naamati-Schneider, Adaya Meirovich

**Affiliations:** ^1^Education and Community, Kinneret College on the Sea of Galilee, Galilee, Israel; ^2^Management of Service Organizations, Hadassah Academic College, Jerusalem, Israel

**Keywords:** future problem-solving, distance learning, eHealth Literacy, health education, higher education

## Abstract

The current study describes the implementation of an online Future Problem Solving (FPS) program in the field of Health education and set out to explore its contribution to students' eHealth Literacy identity, by using two levels of teacher guidance: minimal vs. frequent. FPS was employed in two groups of Health students. In the research group, frequent weekly guidance was provided to the students centered on the enhancement of eHealth Literacy skills, whereas in the control group minimal guidance was offered by the lecturer. Data for the analysis were gathered from 113 Israeli undergraduate students of a Management of Health Service Organizations program, of whom 62 comprised the research group. Data were gathered twice, pre- and post-program implementation from both groups. Findings showed significant differences between the tests only for the research group, with increased levels of eHealth Literacy skills detected between the tests. The perception of the FPS program as meaningful contributed to students' perceived eHealth Literacy skills only in the research group whereas non-significant results were shown for the control group. This study mainly shows that the enhancement of skills in online educational environments requires frequent and personalized guidance. Faculty must recognize the role of the instructor as a facilitator of learning and design successful scaffolding strategies to nurture students' lifelong learning skills during distance learning.

## Introduction

Coping with the wide array of urgent needs raised by the COVID-19 pandemic outbreak requires staff in the healthcare system to demonstrate diverse adaptive capabilities. These include skills such as solving ill-structured problems and digital literacy. These lifelong learning skills are becoming an essential part of the array of tasks that characterize healthcare professionals, and designers of higher education settings must assume an important role in honing these skills by employing innovative instructional approaches (Berkhout et al., 2018).

This study employed online Future Problem Solving (FPS) learning and instruction method aided by technology during an online course. FPS allows students to think creatively and imaginatively to address contemporary challenges that might get worsen in the future (Treffinger et al., 2012). This technique includes identifying and researching a future underlying problem, raising possible solutions to the problem, evaluating them according to different criteria, making an informed choice of solution, developing an action plan, and demonstrating how the solution found will work to solve the underlying problem (Cramond, 2009).

A central twenty first-century skill practiced during FPS is digital literacy, defined as the awareness, attitude, and ability of individuals to appropriately use digital tools and “…to identify, access, manage, integrate, evaluate, analyze, and synthesize digital resources, construct new knowledge, create media expressions, and communicate with others” (Martin, 2005, p. 135–136). The current study presents an intervention practice of FPS program and set out to explore its contribution to students' digital literacy identity in the field of health education. Hence, it focuses attention on eHealth Literacy, succinctly defined as the ability to access medical information from digital sources, evaluate its quality, and apply it in the context of health (Mehoudar, 2014).

Another key issue central to this study is the level of teacher guidance in online courses during the pandemic. Indeed, online courses demand more independent learning, however, many students who participate in such courses experience frustration because they lack the self-directed learning skills required in online courses and are not prepared for isolated learning experiences (Kim et al., 2014). In addition, during the pandemic, studies indicated that students have struggled to manage their studies while dealing with financial and emotional hardships (Nguyen and Balakrishnan, 2020).

Based on these premises, the current quasi-experimental study employed FPS program in two groups of Health students distinguished by the level of teacher guidance: minimal and frequent. In the research group, frequent weekly guidance was provided to the students centered on eHealth Literacy skills enhancement; whereas in the control group, minimal guidance was offered by the lecturer, yet the students were encouraged to consult their difficulties with the teaching staff. The main hypotheses were that the research group's eHealth Literacy skills will be enhanced post-intervention compared to control group data; and that the perception of the intervention as meaningful, namely nurturing future thinking, would contribute to research students' perceived eHealth Literacy ability.

The novelty of this research lies in its initial attempt to show how FPS can be utilized online in Health education, accompanied by effective guidance, and to demonstrate the contribution of this method to students' eHealth Literacy identity. This study might offer an effective instructional e-model for higher education to enhance students' future thinking and digital literacy skills.

## Literature Review

### E-Health Literacy

During the last decade, the concept of literacy has expanded beyond reading and writing, numeracy, and solving problems at the skill level that merely allows proper function in work and society (National Institute for Literacy, 1991; Alt and Raichel, 2020). The definition of literacy has expanded to include a wide variety of skills in various areas with the objective of developing personal knowledge and potential in specific areas such as finance, media science, and medicine (Mehoudar, 2014). During the past few decades, the manner in which people relate to the concept of health literacy has also expanded as a result of environmental changes that have led to additional demands and needs of the population (Nutbeam, 2000; Peerson and Saunders, 2009). The concept of health literacy currently refers to several aspects: The functional aspect—the ability to understand and to make use of health information; the interactive aspect—the ability to develop skills for taking action and interact with health system workers; and the critical aspect—the ability to understand information about social and political factors that influence health and to analyze information in a critical manner. Defining health literacy refers to the aspects of both the individual and the society. For example, understanding medical journals and explaining issues to the public may contribute to the health of individuals but also may have social repercussions in terms of promoting health, cutting down government expenditures, and eliminating inequality in health issues (Mehoudar, 2014).

In light of the processes of digital transformation that characterize the last decade (Topol, 2012; European Commission, 2020), it is now accepted to include eHealth Literacy as part of health literacy. E-Health Literacy refers to the ability to find and understand medical information from digital sources, assess its quality, and apply it in the context of health (Mehoudar, 2014). Tools and questionnaires were constructed to assess the level of online health literacy among the general population. An example of this is the Lily Model that includes several areas of literacy: basic literacy, health literacy, information literacy, media literacy, computer, and scientific literacy (Norman and Skinner, 2006).

Numerous studies have examined the connection between the level of eHealth Literacy and online medicine in relation to variables connected to levels of health and preventing illness. People with high levels of eHealth Literacy reported that they engaged in healthy physical activity and ate a healthy diet (Mitsutake et al., 2016). Good critical eHealth Literacy was found to be a predictor of a healthy lifestyle: responsibility for health, interpersonal support, nutrition, coping with pressure, and physical activity (Yang et al., 2017). A study of medical students revealed that good acquisition of these skills also comprised an effective tool for coping with fear, pressure, and anxiety that arise when providing medical care in stressful and uncertain situations such as the outbreak of the COVID-19 pandemic (Nguyen et al., 2020). People with higher levels of eHealth Literacy were for the most part younger, better educated, and used electronic devices more frequently (Tennant et al., 2015).

On the other hand, low eHealth Literacy in the population was found to be connected with poor health, insufficient understanding of medical conditions, an unhealthy lifestyle, low levels of socio-economic conditions, and lack of education among adults of varied ethnic backgrounds (Mullan et al., 2017). Another study (Diviani et al., 2015) showed that low levels of eHealth Literacy in the population were connected to lower levels of knowledge about illness, more symptoms of illness, less use of health resources, a higher rate of hospitalizations, more frequent use of emergency medicine, and less frequent participation in surveys and use of preventive medicine. Similarly, Sørensen et al. (2015) found that there is a broad range in the levels of health literacy among certain populations including older people, persons with lower levels of education, people who suffer from health problems, members of minority groups who have limited access to resources, and people from low socio-economic backgrounds.

These findings bear far-reaching repercussions upon conditions of disease/morbidity among individuals and the population combined with processes of inundation of information and unreliable medical information on the Internet and social media. These trajectories have made eHealth Literacy one of the most important issues in promoting the health of individuals and of the entire society (Norman and Skinner, 2006; Mehoudar, 2014; Paakkari and Okan, 2020). In addition, the outbreak of COVID-19 throughout the world in 2020 increased the understanding of the issue of eHealth Literacy. It also demonstrated the significance of the need for these skills within the framework of preventing the further spread of the virus and encouraging people to be vaccinated (Paakkari and Okan, 2020). There is no doubt that the processes of educating the population and increasing health literacy skills and online health literacy among the population are extremely important for treatment, prevention, obtaining better cooperation in caregiver-patient relations, and for closing the gaps in the health of the entire society (Mehoudar, 2014).

In addition to these changes and the need to develop eHealth Literacy among the entire population, it is also essential to develop these skills among students of the medical professions and medical staff. This will constitute part of their adaptation to the change processes in the environment and the profession (Mullan et al., 2017). These changes were brought about following the establishment of digital medicine and the implementation of modern technologies that are characteristic of the fourth industrial revolution. Innovative technologies have resulted in rapid changes in numerous areas and made the boundaries between the physical, digital, and biological worlds vague and unclear (Schwab, 2017). These changes have made digital information extremely accessible and there is now a need to process and assess its reliability for both patients and caregivers (Hemmo-Lotem and Shani, 2018). Changes in the paradigm of medical processes are moving in the direction of personalized medicine based on Big Data that has become an essential tool for doctors and patients. These trends are joining the changes in perception of medicine with the prognosis of use of artificial intelligence, the move toward personalized medicine, and an increase in the instances of home hospitalizations as part of the changes in the traditional role of hospitals (Hemmo-Lotem et al., 2021).

These changes emphasize the need to improve eHealth Literacy skills among individuals and medical teams as part of their adaptation to current changes. This process is taking place within the fourth industrial revolution that has repercussions upon the medical world (Schwab, 2017). Improving these skills among medical workers and the general population will improve patients' safety, prevent diseases, promote health, improve doctor-patient communications, create social equality, and eliminate opposition to the change process (Hemmo-Lotem et al., 2021). Honing online health literacy skills improves self-management processes among both caregivers and patients and encourages them to lead processes as part of the changes in health paradigms and the role of doctors and the medical world (Kaper et al., 2019). The changes of the fourth industrial revolution have created a complex reality that requires building multi-dimensional online health literacy abilities and skills among patients and medical staff. Teaching and developing these abilities will enable them to better adapt to health processes in the twenty-first century and to the changing demands of their jobs (Mullan et al., 2017; Hemmo-Lotem and Shani, 2018; Hemmo-Lotem et al., 2021).

### Future Problem-Solving Program

The importance of fostering students' ability to solve future problems had been underscored in educational programs worldwide during the last two decades (Treffinger et al., 2021). These programs use complex, open-ended problems that are based on daily life, relevant to the future, and rooted in social contexts. The problem at the core may address concerns about current trends that may develop and affect the human race in the future, and the solution should suggest changing or adapting society to future situations. That is, the problem must be complex and address social, political issues, business, or technological issues and must take into account future trends that are ingrained in the current era. By this inquiry activity, which invites students to present creative solutions, students are expected to develop skills that they can apply throughout their lives (Main et al., 2019).

FPS program includes six sequential steps (Torrance and Cramond, 2002; Cramond, 2009): (1) identifying challenges in a future problematic or fuzzy situation. In this step, many possible problems might arise from the given situation. Students need to identify several problems related to the situation and find information that might help them understand the issues at hand; (2) selecting an underlying problem to be tackled. Based on the previous step, participants are asked to choose a core problem that, if solved, could immensely contribute to solving the larger situation. This step may include reverting to research to get more information or even limiting or enlarging the focus of the problem statement; (3) producing possible solutions to the underlying problem without judgment. Students are required to create varied, unusual, unlikely, and fantasy solutions for the chosen problem. Such ideas might prove viable or might spur someone else in the group to think of a great idea, “many of our most innovative ideas have come about from what was undoubtedly considered crazy at one time” (Cramond, 2009, p. 10); (4) generating and selecting evaluation criteria for the suggested solutions. In this step, students indicate appropriate criteria by which to evaluate their solutions. Standard criteria can include qualities such as: safe, effective, efficient, possible, legal, ethical, or humane; (5) evaluating and ranking the possible solutions according to the criteria and choosing the best one; (6) developing an action plan, demonstrating how the solution will work to solve the underlying problem. This step requires students to consider how they might implement their solution to effectively solve the problem. A plan must be devised to persuade key stakeholders to adopt the suggested idea.

It should be noted that previous research falls short of assessing the impact of FPS programs on students' eHealth Literacy skills. Yet, several researchers evaluated students' problem-solving skills and digital literacy abilities. For example, Ozdamar-Keskin et al. (2015) assessed digital literacy skills and learning habits of university students enrolled in an open and distance education system. According to their findings, learners reported having problem-solving skills, with only basic competencies of digital literacy skills, and basic knowledge of how to use information and communication technologies.

Other researchers (Quann, 2015; Frank and Castek, 2017; Vanek, 2017) suggested advancing digital literacy through problem-solving activities. These researchers argued that advancing basic digital literacy skills is insufficient, and teachers need to prepare their students to “skillfully use digital tools and develop a discovery and risk-taking mindset toward navigating online” (Frank and Castek, 2017, p. 66). This requires advancing digital problem-solving skills, rather than basic digital skills, which include locating, evaluating, creating, and communicating information to solve ill-structured real-world problems in technology-rich environments (Quann, 2015).

### Teacher Guidance in Online Courses

Teacher guidance has been identified as pivotal in online courses which demand more self-regulated learning. Students who participate in such courses and lack self-directed learning abilities might experience frustration as they are not prepared for isolated learning experiences (Kim et al., 2014). In addition, with the turn to distance learning during the pandemic, students often struggled to manage their studies while dealing with financial and mental health hardships related to the social distancing imposed due to the pandemic outbreak (Nguyen and Balakrishnan, 2020).

Kebritchi et al. (2017) asserted that distance learning changes the role of teachers and the way students learn in higher education. In their study, they synthesized prior studies and provide three major categories relating to online courses: (1) learners' expectations, readiness, identity, and participation in online courses; (2) instructors' issues including changing faculty roles, transitioning from face-to-face to online, time management, and teaching styles; (3) content issues included, among others, the role of instructors in content development, and instructional strategies. Online learning styles and skills are required to successfully participate in online courses, therefore, online instructors should identify and help learners who lack skills, such as technical skills and time management skills (Garcia et al., 2018; Alt and Naamati-Schneider, 2021a). Moreover, learners may feel isolated in online courses. Therefore, teachers should increase students' sense of identity and belonging by encouraging them to affiliate with communities of learning (Koole, 2014).

Effective teaching guidance should include satisfactory faculty-student interactions, setting expectations for interactions and effective communication both between faculty and students and students and their peers, and using diverse e-learning methods and strategies (Kay and Pasarica, 2019). Kebritchi et al. (2017) maintained that the instructor plays the most important role in determining student success in online settings. The mode of communication between faculty is essential for students' success in online learning. This must be personalized including a “personal touch”, and identification of trends occurring in the online class to adapt the teaching style accordingly. Effective instructors of online classes should strive to create a community of learners, and provide a safe environment where students feel free to share their values and ideas.

Similarly, Freeman and Jarvie-Eggart (2019) asserted that teacher-learner interaction is a core element of any online course. The instructor should be actively present in online learning and promote regular and substantive interaction with the learners. A strong sense of instructor presence improves student outcomes in online courses. Recent studies (De Leeuw et al., 2019; Regmi and Jones, 2020) in health education assessed the factors affecting e-learning in health education and outcomes and methods used to evaluate medical education e-learning by performing a systematic literature review. For example, Regmi and Jones (2020) identified several factors that impact the interaction and collaboration between learners and facilitators, such as learners' motivation and expectation and lack of IT skills. Yet, empirical evidence of how teachers can nurture their students' digital literacy competence via online courses is still quite limited (Alt and Raichel, 2020).

### This Study

The literature review shows how instructional approaches, such as problem-based learning, were used to promote digital literacy skills (e.g., Ozdamar-Keskin et al., 2015), however, these skills were not assessed in the context of FPS programs. The review also underscores the central role the teacher plays in online courses (Kebritchi et al., 2017). In accordance with the above-surveyed studies, the current quasi-experimental research employed a FPS program in two groups of Health students. In the research group, frequent weekly guidance was provided to the students centered on digital literacy skills enhancement; whereas in the control group, minimal guidance was offered by the lecturer, yet the students were encouraged to consult with the teaching staff. Accordingly, the following research questions and hypotheses were formulated:

(Q1) Which teaching guidance style (minimal vs. frequent guidance during the FPS program) would be more effective in promoting Health students' eHealth Literacy? To assess the effectiveness of teaching styles in this aspect, a quasi-experimental, pre/post-test design was used to examine students' perceived eHealth Literacy before and after the program implementation. It was expected that participants who received frequent assistance from the teacher (research group) would report attaining higher levels of eHealth Literacy skills, following the implementation of the FPS program compared to its onset, than the control group (H1).

(Q2) How the perception of the FPS program as meaningful (in terms of advancing future thinking) would impact students' reported eHealth Literacy skills? It was expected that the research group students would tend to attribute the increase in eHealth Literacy skills to the program (H2). This trajectory was also expected in the control group, yet we anticipated a relatively lower impact of the program on the eHealth Literacy skills compared to the research group (H3).

## Method

### Participants

Data for the analysis were gathered from 3rd-year 113 Israeli undergraduate students of a Health Management program (covering patient-doctor relations, quality of service in the healthcare system, and ethics and patient rights), of whom, 62 comprised the research group. Participants were enrolled in two courses dealing with quality in health systems. Both groups (research and control) were taught by the same instructors. Table 1 details the research and control students' age, gender, ethnicity, and socioeconomic status (SES) derived from the educational attainment of the students' parents defined on a six-level scale: 0 = *lack of education*, 1 = *elementary school*, 2 = *high school*, 3 = *BA degree*, 4 = *MA degree*, 5 = *doctoral degree*. Non-significant between-group differences were found on all the measured variables. Researchers emphasized prior to obtaining consent that the questionnaires were both anonymous and voluntary. Finally, participants were assured that no identifying information about the courses would be processed. The research was approved by the college's Ethics Committee.

**Table 1:** Student characteristics (research and control groups).

	**Research group**	**Control group**
Age	Mean 24.33 (*SD* = 5.77)	Mean 27.28 (*SD* = 7.90)
Gender	80.8% females	71.3% females
Mother educational attainment	Mode 3 (high school)	Mode 4 (BA degree)
Father educational attainment	Mode 3 (high school)	Mode 3 (high school)
Ethnicity	61% Jews 36% Minorities 3% unidentified	78.7% Jews 21.3% Minorities

### Measurements

#### eHEALS Scale

This eight-item scale (Norman and Skinner, 2006) was designed to measure students perceived digital skills at using information technology for health. Items such as “I know how to find helpful health resources on the Internet” were scored on a five-point Likert-style format scale (from 1= *strongly disagree* to 5= *strongly agree*). Cronbach's alphas ranged from 0.88 to 0.92.

#### Future Thinking

Based on the theoretical framework, this scale was constructed for the purpose of the current study. This six-item scale corresponds to the six steps of the FPS program (Torrance and Cramond, 2002). The participants were asked to indicate the extent to which the program raised their level of awareness of social problems that might arise in the future and ways to solve them. Items such as “I think about major social issues that may arise in the future” were scored on a six-point Likert-style format scale (from 1= *never* to 6= *always*). Cronbach's alphas ranged from 0.86 to 0.93.

### Procedure

The research and control groups were enrolled in a 3-month FPS program, including the following six steps (Torrance and Cramond, 2002; Cramond, 2009).

#### Step 1

The step included introducing a future broad challenge and breaking it down into a number of problems. The broad challenge related to a future hypothetical possibility of conducting long-distance meetings in the event of a continuous pandemic that would prevent face-to-face meetings for treatment. The students were “informed” that the COVID-19 pandemic is here to stay. In the challenge description provided to the students, rising morbidity rates were indicated, accompanied by a lack of an effective vaccination for newly detected mutants. This crisis led to the dismal state of the health system and hospitals manifested in a lack of manpower and medication. This means, as was depicted in the problem statement, that there is no quick return to the routine that we knew which allowed patients to be admitted to hospitals and clinics. As a result, health organizations are instructed to switch to receiving patients online and provide remote services only. Patients in need of hospitalization will be hospitalized in remote and isolated conditions until further notice. This implies that aside from hospitalization in extreme cases, patients would not come to the clinics or hospitals for treatment, and doctors would have to administer diagnosis and treatment from a distance.

After the introduction of the broad challenge, students were divided into groups of four where they identified specific problems that might stem from the general future challenge and related them to patient-doctor relations, quality of service ethics, and patient rights. In other words, they were asked to indicate what problems might arise from online service and medical care rather than prolonged face-to-face contact. In accordance with the course content, the following areas could have been considered in this stage: technological problems, inability to communicate, inadequate conditions for diagnosis, lack of cooperation with patients, application problems among physicians, application problems among certain population groups such as the elderly, difficulties in forming patient-therapist personal relationships and the like.

This step entails early research by each group to understand the challenge, and its embedded, related problems (Cramond, 2009). The groups thought about challenges such as the response of various population groups that have varying levels of digital abilities, the high cost of adapting the system, difficulties and opposition to adapt on the part of doctors and the medical staff, problems establishing intimate contact with the medical staff, and other issues.

An online meeting was conducted with the research group in which an example of the challenge was presented by the lecturer. Students were instructed to search for materials addressing this challenge. The control group received only written instructions.

#### Step 2

Each group selected one problem related to one of their specific challenges and formulated it as a question. For example: How to solve the problem of access to online medical services among the elderly or among other sectors with limited digital skills; or how to improve intimacy and trust between a caregiver and a patient in online medical appointments.

#### Step 3

At this step, each group produced four solutions to the problem formulated in Step 2 and conducted a brainstorming session during which they were instructed to imagine any solution that came to mind, regardless of rationale or feasibility. To formulate solutions, groups accessed more information by reading materials related to the course content. In relation to eHealth Literacy, the research group students were guided by the lectures who provided weekly sessions. These were mainly centered on honing digital eHealth Literacy skills needed to substantiate their solution to the future problem. The online guidance included (1) instructions and examples of how to navigate digital media, access information by locating and sharing materials; (2) how to analyze messages in a variety of forms, obtain information critically, identify the source of information, and evaluate the quality and credibility of the content; (3) ways to create content in a variety of forms, which enable making use of language, images, sound, and new digital tools and technologies, and effectively collaborate to construct new knowledge or digital artifacts using technology and media. Students in the control group received an asynchronous session regarding eHealth Literacy and were encouraged to seek the instructor's feedback via email or zoom meetings, yet synchronous lessons were not scheduled for this purpose due to a lack of institutional resources.

#### Step 4

This step included devising criteria to evaluate the solutions the students raised. After small group discussions, a Google Doc was created in which each group could upload five ideal criteria. Next, the lecturer with the help of student suggestions selected the five most appropriate criteria to assess the solutions. The criteria were: the solution is creative, easy to adapt, applicable, inexpensive in terms of manpower and funds, safe, has a long-term effect, does not require complicated technology, and contributes to the entire society.

#### Step 5

Each group evaluated their four solutions according to the criteria using a grid that indicated the quality of each criterion on a Likert-type score ranging from 1 = *not at all* to 6 = *absolutely*.

#### Step 6

Finally, each group devised a plan to pitch their desired solution. In this plan, they specified their suggested steps for implementation and described how the solution might work, who could help them implement it, who might oppose it, and how such challenges could be mitigated. The students had to present their action plan in a plenary meeting using online platforms such as “Thinglink”. In terms of eHealth Literacy, they constructed new knowledge by creating media expressions and communicating them with others. Exemplary solutions were: developing biometric security measures for preserving medical confidentiality, creating safe areas in open spaces for conducting online discussions, preparing a support system for the elderly population based on one-on-one solutions within distancing regulations for instructing them in medical literacy and increasing their digital abilities, developing equipment for long-distance diagnosis and treatment, etc.

The different treatments given to the research and the control groups stemmed from the limited institutional resources available during the pandemic outbreak. This study was designed as pilot research with the goal of evaluating the impact of minimal vs. frequent guidance in an online FPS program on students' eHealth Literacy skills. The objective was to implement the program more widely depending on this study's results.

### Data Analysis

Analysis of variance was applied to allow the characterization of differences between the pre- and post- interventions within the research and control groups. Data were also analyzed using Partial Least Squares Structural Equation Modeling (PLS-SEM; Hair et al., 2017), advised for situations where theory is less developed and the primary objective of applying structural equation modeling is the prediction of target constructs.

### Findings

The first research question related to the teaching guidance style (minimal vs. frequent guidance during an FPS program). It was postulated that participants who received frequent assistance from the teacher (research group) would report attaining higher levels of eHealth Literacy skills, following implementation of FPS compared to the onset of the program, than the control group.

**Figure 1:**
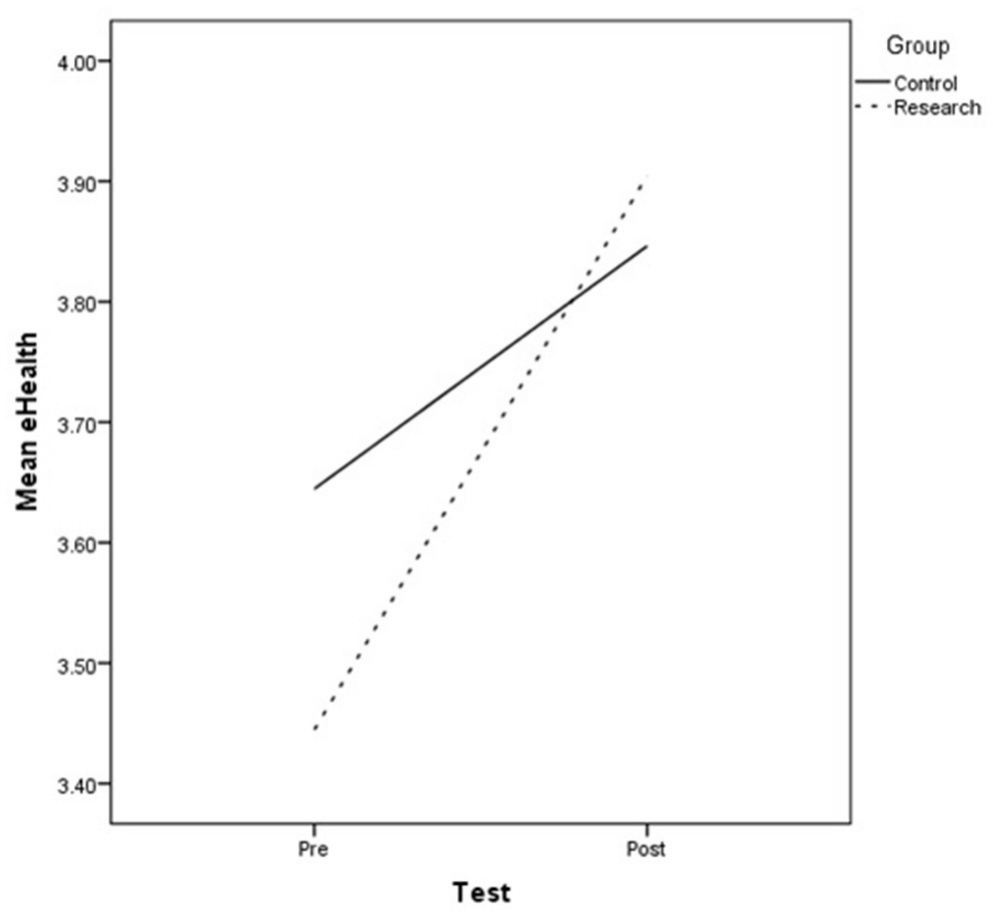
Within-group differences between the tests.

A univariate analysis was applied to allow the characterization of differences between the pre- and post- test in each group (research/control) on eHealth Literacy skills. As shown in Figure 1, the analysis showed significant differences between the tests [*F*_(1, 102)_ = 12.664*, p* < 0.01, η^2^ = 0.110] for the research group, with increased levels of eHealth Literacy skills detected between the tests (pretest *M* = 3.44 *SD* = 0.68; posttest *M* = 3.90 *SD* = 0.59). Non-significant results were shown between the tests for the control group [*F*_(1, 92)_ = 1.993*, p* > 0.05, η^2^ = 0.021]. H1 was corroborated.

The second research question dealt with students' perception of the FPS program as a precursor of eHealth Literacy skills. It was postulated that the research group students would tend to attribute the increase in eHealth Literacy skills to the efficiency of the program (H2).

**Figure 2:**
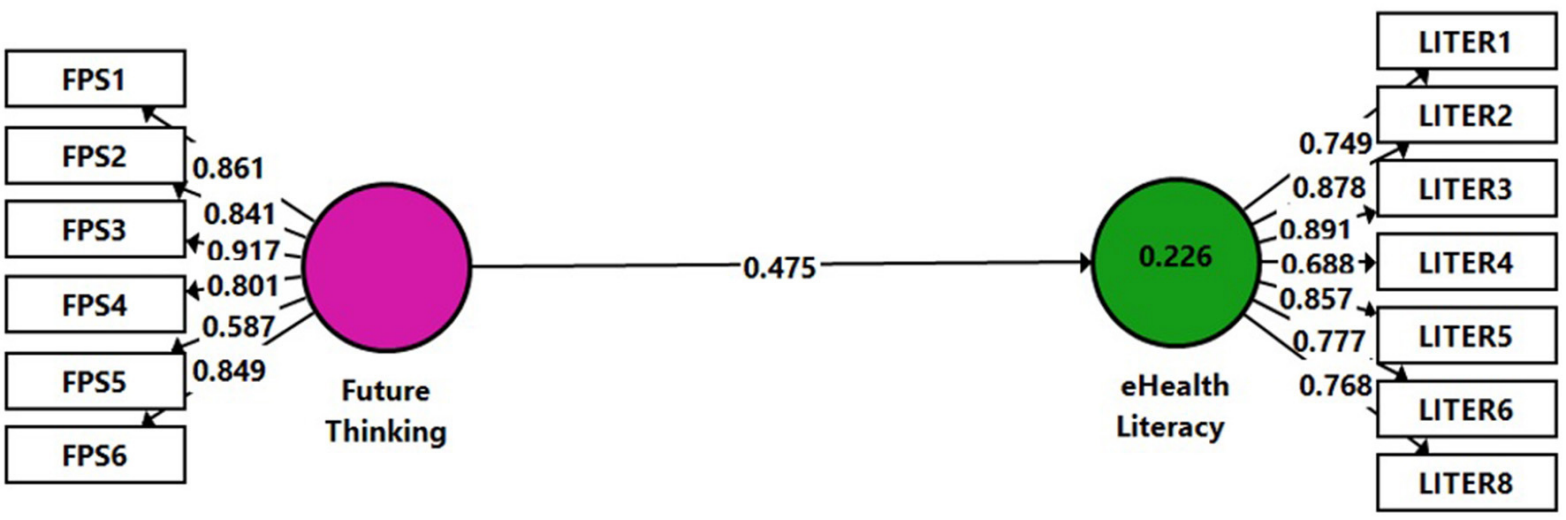
Model 1. Analysis results of the examination of H2 by SmartPLS.

To assess H2, Model 1 (Figure 2) was constructed. This path model includes two constructs, represented in the model as cycles: Future Thinking as an independent variable and eHealth Literacy as a dependent variable. The indicators are the directly measured proxy variables, represented as rectangles (one eHealth Literacy item was omitted due to a low loading result <0.40). Relationships between the constructs as well as between the constructs and their assigned indicators are shown as arrows. In PLS-SEM, single-headed arrows, shown between the constructs, are considered predictive relationships and, with strong theoretical support can be construed as causal relationships.

The results showed a significantly high positive connection between the variables (β = 0.475, *p* < 0.01), namely, the Future Thinking independent variable was positively and moderately connected to the eHealth Literacy dependent variable among the research group. This confirms H2. The model evaluation included the examination of the coefficient of determination (*R*^2^) value. *R*^2^ (0.226) can be considered moderate (Hair et al., 2017). Finally, the blindfolding procedure was used to assess the predictive relevance (*Q*^2^) of the path model. Values larger than 0 suggest that the model has predictive relevance for the endogenous construct (Hair et al., 2017). The *Q*^2^-value indicated for our model was 0.092.

**Figure 3:**
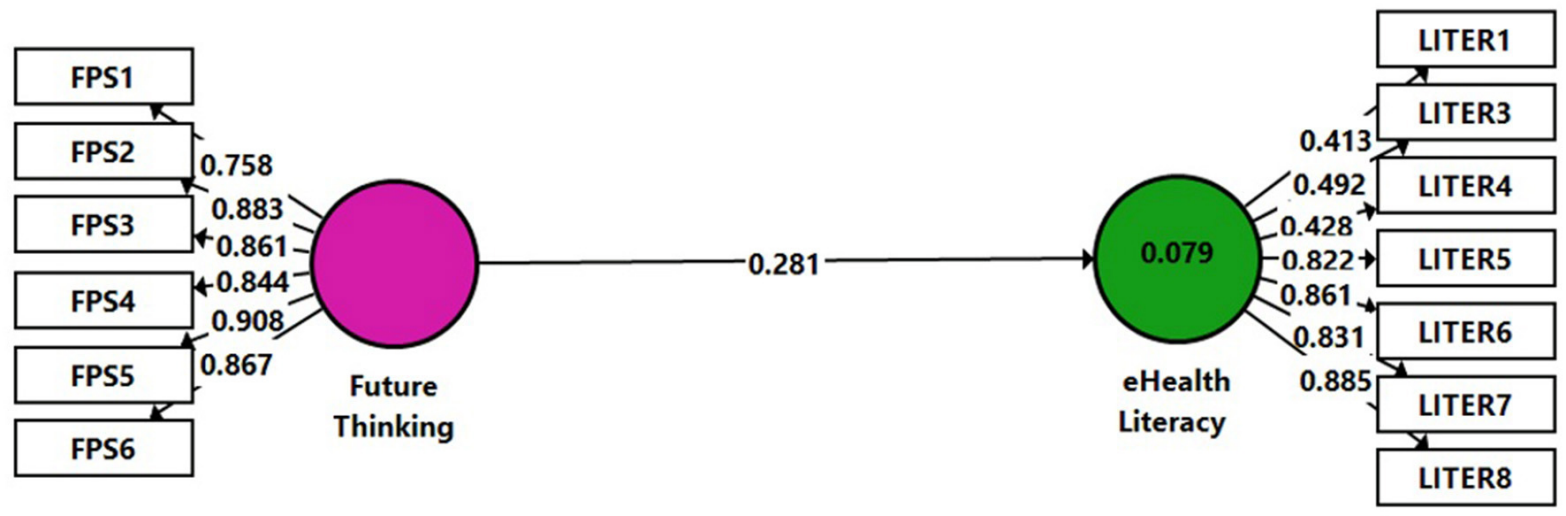
Model 2. Analysis results of the examination of H3 by SmartPLS.

According to H3, a positive link was expected to be obtained in the analysis of the control group's data, with a relatively lower impact of the program on the skills compared to the research group. The same model as in Figure 2 was used to check H3 with data gathered from the control group (Figure 3, Model 2). A non-significant result was obtained between the variables (β = 0.281, *p* > 0.05), namely, the connection between the Future Thinking independent variable and the eHealth Literacy dependent variable was found non-significant for the control group. Hence H3 was confirmed. *R*^2^ (0.079) can be considered very low (Hair et al., 2017). The *Q*^2^-value indicated for our model was close to zero 0.001.

## Discussion

In the current study, an FPS program was employed in two groups of Health students distinguished by the level of teacher guidance: minimal vs. frequent. It mainly sought to assess the program's impact on Health students' eHealth literacy skills. The first research question related to the potential effect of teaching guidance styles, minimal vs. frequent guidance, practiced during the FPS program, on students' perceived levels of eHealth Literacy skills. According to the findings, increased levels of eHealth Literacy skills were detected after the intervention in the research group compared to its beginning, whereas non-significant results were shown between the tests for the control group. In addition, students in the research group tended to attribute the increase in eHealth Literacy skills to the efficiency of the program and its ability to raise their level of awareness of social problems that might arise in the future and ways to solve them. This trajectory was found non-significant in the control group.

It appears that the manner of guidance and the role of the lecturer were essential for developing eHealth Literacy skills among the students. The research group received extensive guidance through workshops and active learning to develop their abilities to locate and use material within the framework of eHealth Literacy and to practice and develop those abilities collaboratively. The online instruction that the control group received consisted of only general guidelines about the requirements of the assignment in a non-synchronized manner with no active guidance from the lecturer.

The findings of this study emphasize the importance of the lecturer's mediation in the form of guidance while devoting time to the processes of developing eHealth Literacy abilities among students in the health professions. They also emphasize the changes that need to be implemented in the role of academia in medical education. These changes involve training graduates to adapt to the changing environment of the medical world and the demands of the health care system and working world of the twenty-first century. Updated instructional approaches are required to support the adaptation to the changes that the health care system is undergoing due to the fourth industrial revolution and its long-term impact upon numerous areas of life (Mullan et al., 2017; Schwab, 2017). This is part of the adaptation process of the health care system and its workers to environmental changes and to digital transformation processes that have occurred during the past decade, which were accelerated by the COVID-19 pandemic and its repercussions (Alt and Naamati-Schneider, 2021a).

These changes necessitate an updated mission statement of the academia in line with the overall trend that marks the transformation of its role from an agent of knowledge to a leader of change and an agent of culture, whose responsibility is to develop abilities that are suitable to the twenty-first century and its demands. The role of academia and its institutions was previously perceived as providing of knowledge. Today, however, their role is perceived as to teach and develop new abilities among students and to prepare them for today's work market (Alt and Naamati-Schneider, 2021b). They must be capable of solving real problems in real-time and synthesizing their existing personal knowledge while dealing ceaselessly with the challenges of the new, constantly changing world (El-Benny et al., 2021). The need has arisen within the framework of these abilities for a high level of literacy and extensive online medical literacy. This is particularly true in the health professions that promote the ability to improve treatment and management while improving self-management processes among both patients and caregivers (Kaper et al., 2019). The findings of the current study show that developing lifelong learning skills requires mediation and direction by lecturers in an online environment.

### Limitations and Future Directions

The present work features limitations and directions for future research that warrant mention. The study assessed students' perceptions of their awareness of FPS and digital literacy skills using a self-reporting survey. Some studies find substantial biases in self-report measures and strong divergence between subjective and objective assessments thus data gained by such measures should be interpreted cautiously (Bowman, 2010). However, it is noteworthy that students' perceptions, attitudes, and beliefs play a central role in their learning and are related to motivation to engage in a specific learning activity (Bandura, 1997). In the context of the current study, for example, student reports on having increased awareness about social future problems may drive their future actions. Hence, measuring student perceptions may help to understand their role in facilitating FPS programs and evaluating their impact. Lastly, students in the control group received an asynchronous session regarding eHealth Literacy and were encouraged to seek the instructor's online feedback. In contrast to the research group, synchronous lessons were not scheduled for the control group. It is plausible to assume that these different modes of communication (synchronous vs. asynchronous) affected the results of this study. Future studies should consider controlling this variable to better understand the effect of FPS programs on students' digital skills in distance learning different modes of interaction.

## Conclusions and Implications

This research provides a useful constructivist pedagogical tool of FPS accompanied by effective guidance and illustrates how this method might be utilized online to advance students' eHealth Literacy skills. Whereas, previous research assessed students' problem-solving skills and digital literacy abilities, suggesting that learners possess only basic competencies of digital literacy skills, and basic knowledge on how to use information and communication technologies (Ozdamar-Keskin et al., 2015; Quann, 2015; Frank and Castek, 2017; Vanek, 2017), our study suggests advancing digital skills by using a well-structured pedagogical method of FPS.

**Figure 4:**
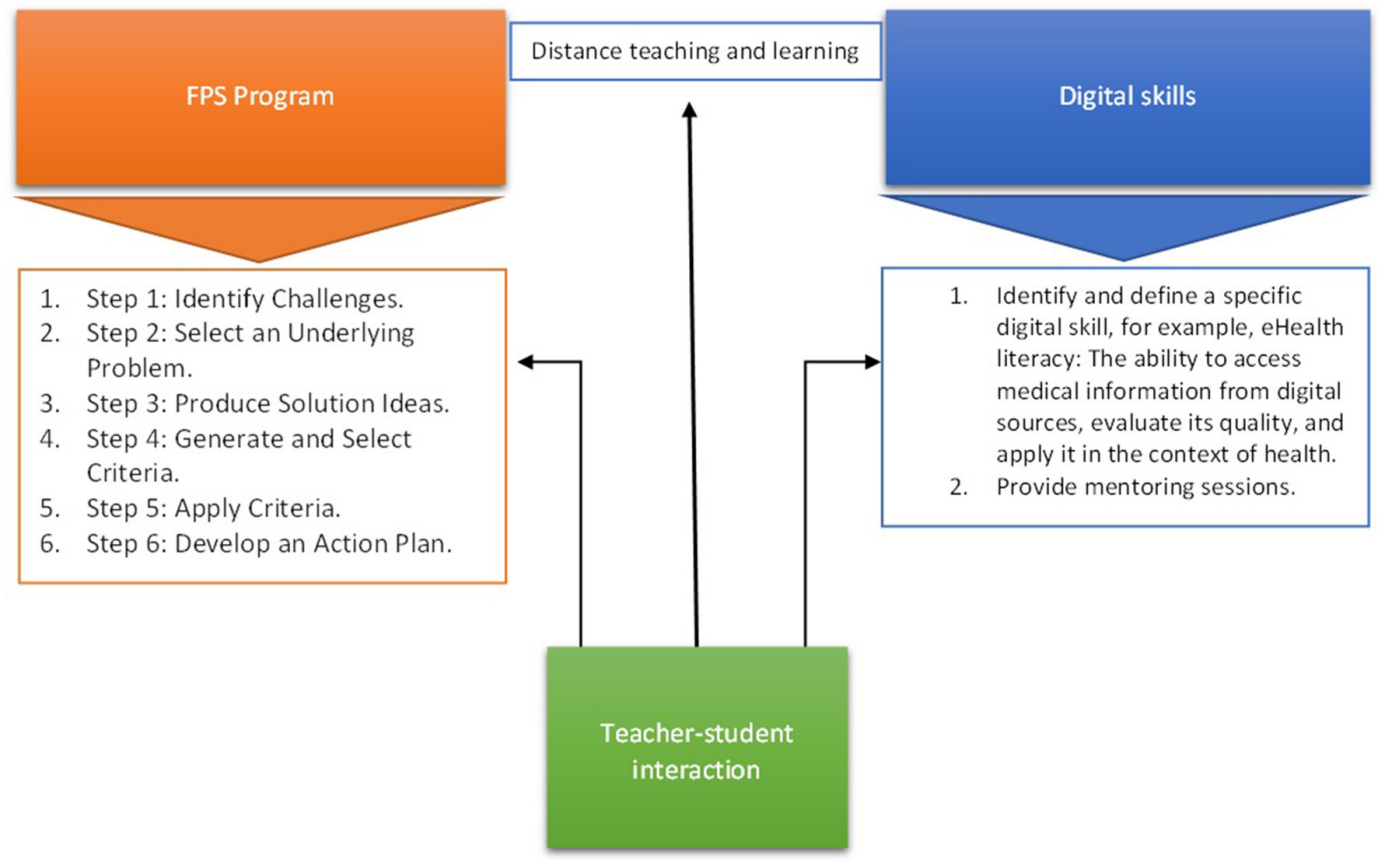
Model 3. Suggested model for the implantation of online FPS program aimed at nurturing students' digital skills.

Based on our findings, Model 3 (Figure 4) is suggested for the implantation of an online FPS program aimed at nurturing students' digital skills. The model includes the six FPS steps (Cramond, 2009), however, in addition to previous FPS programs, digital literacy is specified as a learning outcome. The “teacher-students interaction” addresses three components (from left to right): 1. FPS program is guided by the teachers (e.g., providing examples of the challenges); (2) modes of communication in distance learning that enable effective teacher–student interactions are applied; (3) teachers identify and define the digital skill as a learning outcome, and provide mentoring sessions centered on honing digital skills (e.g., ways to create content in a variety of forms, which enable making use of new digital tools and technologies). Based on our results, it is suggested that teachers should consider raising the quality of their online courses by addressing critical issues such as communication with students (Limperos et al., 2015). Their availability remains an area to be addressed and might determine the quality of their teaching and learning outcomes. In line with previous research, effective online teaching should include effective teacher–student interactions, setting expectations for interactions both between faculty and students and among students (Kebritchi et al., 2017). It seems that the quality of the interaction might determine student success in online courses in terms of achieving designated learning outcomes. To this end, faculty should be provided with appropriate guidance regarding the ways of e-communication available to successfully engage with students, that enables shared communities of learners.

Other suggestions for improving online teaching effectiveness are to tailor constructivist learning methods and strategies to online teaching. Such methods enable interaction and collaboration with students to support their higher-order thinking abilities and other important twenty first-century learning outcomes such as digital skills. Methods such as FPS engage students with thought-provoking problems and spur them to collaboratively offer viable solutions in a safe environment. Teachers might benefit from having training sessions on how to leverage advanced online pedagogical methods to advance their students' lifelong learning skills.

## Data Availability Statement

Datasets are available on request to the corresponding authors.

## Ethics Statement

The studies involving human participants were reviewed and approved by Kinneret Academic College. The patients/participants provided their written informed consent to participate in this study.

## Author Contributions

DA: conceptualization, data curation, writing-original draft preparation, and writing-reviewing and editing. LN-S: data curation, methodology, writing-original draft preparation, and reviewing and editing. AM: data curation, writing-original draft preparation, and reviewing and editing. All authors contributed to the article and approved the submitted version.

## Conflict of Interest

The authors declare that the research was conducted in the absence of any commercial or financial relationships that could be construed as a potential conflict of interest.

## Publisher's Note

All claims expressed in this article are solely those of the authors and do not necessarily represent those of their affiliated organizations, or those of the publisher, the editors and the reviewers. Any product that may be evaluated in this article, or claim that may be made by its manufacturer, is not guaranteed or endorsed by the publisher.
